# Gonococcal Epididymitis in the Soldier

**Published:** 1919

**Authors:** 


**Gonococcal Epididymitis in the Soldier.** By Capt. A. H. Peacock. *Northwestern Med.* October, 1918.

The author reviews briefly the pathology and symptoms of gonococcal epididymitis. He emphasizes the need of surgical treatment of the condition in soldiers, because of the frequent relapses that occur when men are returned to duty. After a few days in bed, nearly complete resolution may take place, but when the man takes up full active duty and drill, he is returned to the hospital in a few days, with a relapse of the infection. Or if put on special duty, if he suffers any physical strain, the result is the same. It was these relapses that suggested the surgical treatment to eradicate the time infection thoroughly, relieve pain immediately, and shorten the time in the hospital.

The operation for acute cases is epididymotomy, and for chronic cases epididymectomy. General anesthesia is used on account of the marked tenderness. Nitrous oxide preferred by the author.

The testis is held firm and turned on its upper pole so that the most prominent part of the swelling is upward. After incising the skin and deep fascia, the epididymis is pressed up through the incision. In acute cases it is split from end to end, and the small abscess usually present sought and opened; the cut edge of the epididymis is sewed to the skin wound to keep it open and preserve drainage; plain gauze is packed in the wound. The wound is dressed and the testicles laid on a bridge of adhesive plaster from thigh to thigh. The wound heals in from 10 days to two weeks, when the soldier may be returned to duty.

In chronic cases, the diseased portion is dissected down to the digital fossa, and the denuded base drawn together by fine plain gut suture. The various sheaths are drawn together again and the wound closed without drainage. The patient is out of bed in a week's time. A large series of cases have been treated by this method with excellent results.

